# Ultra-trace ^239^Pu and ^240^Pu determination in soils using optimized leaching, vacuum-assisted TEVA separation, and isotope dilution ICP-MS

**DOI:** 10.1007/s00216-026-06614-y

**Published:** 2026-06-12

**Authors:** Gerald Dicen, Floriane Guillevic, Judith Kobler, José A. Corcho Alvarado, Stefan Röllin, Olivier Evrard, Christine Alewell

**Affiliations:** 1 Environmental Geosciences, Department of Environmental Science, University of Basel, Bernoullistrasse 30, 4056 Basel, Switzerland; 2 Spiez Laboratory, Federal Office for Civil Protection, Austrasse, 3700 Spiez, Switzerland; 3 Laboratoire Des Sciences du Climat Et de L’Environnement (LSCE/IPSL), Unité Mixte de Recherche 8212 (CEA-CNRS-UVSQ), Université Paris-Saclay, F-91191 Gif-Sur-Yvette, France

**Keywords:** Plutonium isotopes, Ultra-trace analysis, Tracer, ICP-MS, TEVA extraction chromatography, Radiochemical separation

## Abstract

The determination of ultra-trace ^239^Pu and ^240^Pu isotopes such as those in the Southern Hemisphere remains a challenge in soils. Most techniques have been developed for relatively higher Pu activity concentrations, which are not suitable for soils with low Pu content. This study aimed to develop an optimized leaching and separation procedure for soils collected from a subtropical catchment in Brazil, and to evaluate its performance against a fusion digestion procedure with ICP-MS. We carried out multiple adjustments to an established procedure such as increasing the acid volume in the TEVA (TEtraValent Actinides and technetium) resin separation steps, using Fe(II) for redox stabilization, reducing sample mass, and increasing the flow rate through a vacuum box. While Pu activities and ratios were generally consistent across methods, our results revealed that using Fe(II) and increasing the acid volume in the separation step with an increased flow rate led to better method performance. The optimal procedure, named the “PULSE method” (Plutonium extraction from Soils at Low concentrations with high prEcision), achieved a high Pu recovery rate of 69 ± 8% and a mean ^238^U separation factor of 1.29 × 10^4^. Measurement uncertainties were kept below 10% and the detection limits for ^239^Pu and ^240^Pu amounted to 0.0027 Bq/kg and 0.0008 Bq/kg, respectively. Thus, the PULSE method is a practicable and scalable method that allows for a reliable determination of Pu isotopes, which are considered long-term alternative tracers to shorter-lived radionuclides like ^137^Cs for soil and sediment redistribution processes.

**Supplementary Information:**

The online version contains supplementary material available at https://doi.org/10.1007/s00216-026-06614-y.

## Introduction

Fallout plutonium has gained global attention not only because of the health risks associated with this radionuclide but also for its roles as an essential tracer of geomorphological processes and in dating of environmental archives. Plutonium isotopes, specifically ^239^Pu (t_1/2_ = 24,110 a) and ^240^Pu (t_1/2_ = 6,563 a), have been successfully used as an alternative or additional soil erosion tracer to other fallout radionuclides (FRN) such as ^137^Cs (t_1/2_ = 30 a), ^210^Pb (t_1/2_ = 22 a), and ^7^Be (t_1/2_ = 53 d). This is due to their locally more homogenous spatial atmospheric deposition and thus distribution compared to other FRN tracers, which satisfies the spatial variability criteria for erosion assessment [1–3], as well as to their relatively longer half-lives that allow for their applicability over the next few centuries. The ^240^Pu/^239^Pu isotopic ratios are also distinct depending on the fallout source, whether they originate from the different nuclear weapons testing or nuclear power plant accidents that occurred during the last several decades around the world, thereby providing insights into possible inputs of other fallout sources [4–6]. For example, global fallout resulting from nuclear weapon testing dominated by the USA and the former Union of Soviet Socialist Republics tends to have higher ^240^Pu/^239^Pu atomic ratios than the fallout associated with French or British nuclear tests [4, 7]. Fallout ^239^Pu and ^240^Pu have been widely recognized as reliable time markers for the mid-twentieth century, associated with the onset of the Great Acceleration, and were proposed as the primary “golden spike” candidate for the Anthropocene epoch, a designation that, despite extensive scientific support, was not formally adopted by the geological community [8–11].

Both ^239^Pu and ^240^Pu decay by emitting α particles with similar energies (5.105, 5.144, and 5.156; and 5.124, and 5.162 MeV, respectively) and were traditionally measured together by alpha spectrometry and commonly reported as a sum of the two isotopes (^239+240^Pu). In contrast, accelerator mass spectrometers (AMS) are able to measure each isotope individually regardless of sample matrix components, although this possibility comes with a high cost and long measurements times and thus low sample throughput. Advancements in mass spectrometric techniques such as ICP-MS, which are now more widely available than AMS, have now made the cost-effective measurement of each isotope possible [12–14]. Detection limits have also been considerably lowered to a few tens of mBq/kg, depending on the sample mass used [15], allowing for the analysis of plutonium at low environmental levels. Still, Pu needs first to be extracted from the soil to be analyzed and then to be separated from other actinides such as ^238^U, which forms isobars such as ^238^UH^+^ and ^238^UH_2_^+^ and a tailing in ICP-MS and cannot be discriminated from ^239^ and ^240^Pu peaks [16, 17]. Furthermore, U is normally found between 6 and 9 orders of magnitude higher in concentration than Pu in soils [18], and 1 ng/g of ^238^U detected in the final sample solution could lead to 0.03–0.01 fg/g false Pu detection [16]. Thus, an effective extraction and separation technique that achieves a ^238^U separation factor of at least 10^4^ is needed to reduce uncertainties, especially for soils with low Pu content < 0.1 Bq/kg.

A combination of different techniques for the extraction and chemical separation of radionuclides has been developed to rapidly extract plutonium from different sample matrices using various analytical equipment [19–23]. The proposed leaching and separation procedure of Ketterer et al. [21] is the most straightforward method measuring Pu isotopes in soils [2, 5, 24]. However, this leaching and separation procedure, along with other rapid extraction and separation techniques, was developed for and applied to Northern Hemisphere samples where concentrations of Pu > 1 Bq/kg in the topsoil can be expected. Thus, it cannot be easily applied to soils showing lower activities, as typically most of the soils from equatorial regions and the Southern Hemisphere [4]. To the best of our knowledge, only Dowell et al. [25] reported a method applicable to soils with an ultra-low Pu activity (< 0.1 Bq/kg), in this case for samples from Kenya. This was achieved by using 50 g of soil samples extracted with concentrated HNO_3_ at 1:2 soil-to-acid ratio, separated using TEVA columns, and analyzed using ICP-MS/MS. An ICP-MS/MS combined with O_2_ as the reaction gas is able to significantly suppress ^238^U interferences within the instrument resulting in better Pu detection [26]. While the method proposed by Dowell et al. [25], modified from the proposed procedure of Ketterer et al. [21], has been significantly optimized with less reagents and a lighter chemical procedure, it may not be applicable when the sample quantity is limited, and an ICP-MS/MS is unavailable. Additionally, the procedure still uses a high volume of acid to compensate for the large sample mass, and that increases the cost and the environmental impact of the analysis. Accordingly, the current available techniques for ^239^Pu and ^240^Pu remain less practicable for high-volume routine analyses of soils with low Pu activity, which is a challenge primarily for the investigation of soil and sediment samples from the equatorial and Southern Hemisphere regions.

Existing methods therefore fundamentally lack the ability to handle low amounts of sample (e.g., ~ 5 g) while employing simple procedures and achieving high measurement efficiency. In this study, the procedure of Ketterer et al. [21] was modified to come up with practicable and scalable methods for ultra-trace-level Pu measurements in soils developed through the use of Ferralsols collected from a subtropical catchment in Brazil. Ferralsols are highly weathered soils rich in iron oxides and clay minerals. Since Pu has been shown to rapidly attach to common mineral surfaces and form complexes with organic matter [12, 27], Ferralsols provide favorable sorption sites for Pu. Thus, Ferralsols are ideal for testing Pu extraction and separation methods. The methods were designed to use a minimal amount of sample thereby also requiring only minimal acid volume for leaching and the subsequent separation of Pu from other actinides. Our aim was (1) to test leaching procedures suitable for Ferralsols; and (2) to evaluate the performance of these procedures against a fusion digestion procedure.

## Materials and methods

### Reagents, resin, reference material, and instrumentation

All solutions were prepared using very-large-scale-integration (VLSI) grade nitric HNO_3_ and hydrochloric HCl acids (Carl ROTH, Germany); analytical grade sodium nitrite NaNO_2_ (VWR Chemicals, US) and ammonium iron (II) sulfate hexahydrate (NH_4_)_2_Fe(SO_4_)_2_⋅6H_2_O or Mohr’s salt (ThermoFisher Scientific, USA); and ultrapure water from a PRELAB flex Milli-Q water-purification system (ELGA LabWater, Veolia Water Technologies, UK). TEVA (TrisKem, France) extraction chromatography resin in pre-packed 2 mL columns with 100–150 µm particle size was used to separate Pu isotopes from other actinides in the samples. The standard reference material 4334I ^242^Pu (NIST, Maryland, USA) in HNO_3_ solution, diluted gravimetrically to a concentration of 10 pg/mL, was used for checking the radiochemical yield of the different methods. Mass spectrometric measurements were performed using an Element 2 double-focusing sector field Inductively Coupled Plasma Mass Spectrometry(sf-ICP-MS) (Finnigan MAT, Bremen, Germany).

### Sample description and preparation

**Fig. 1:**
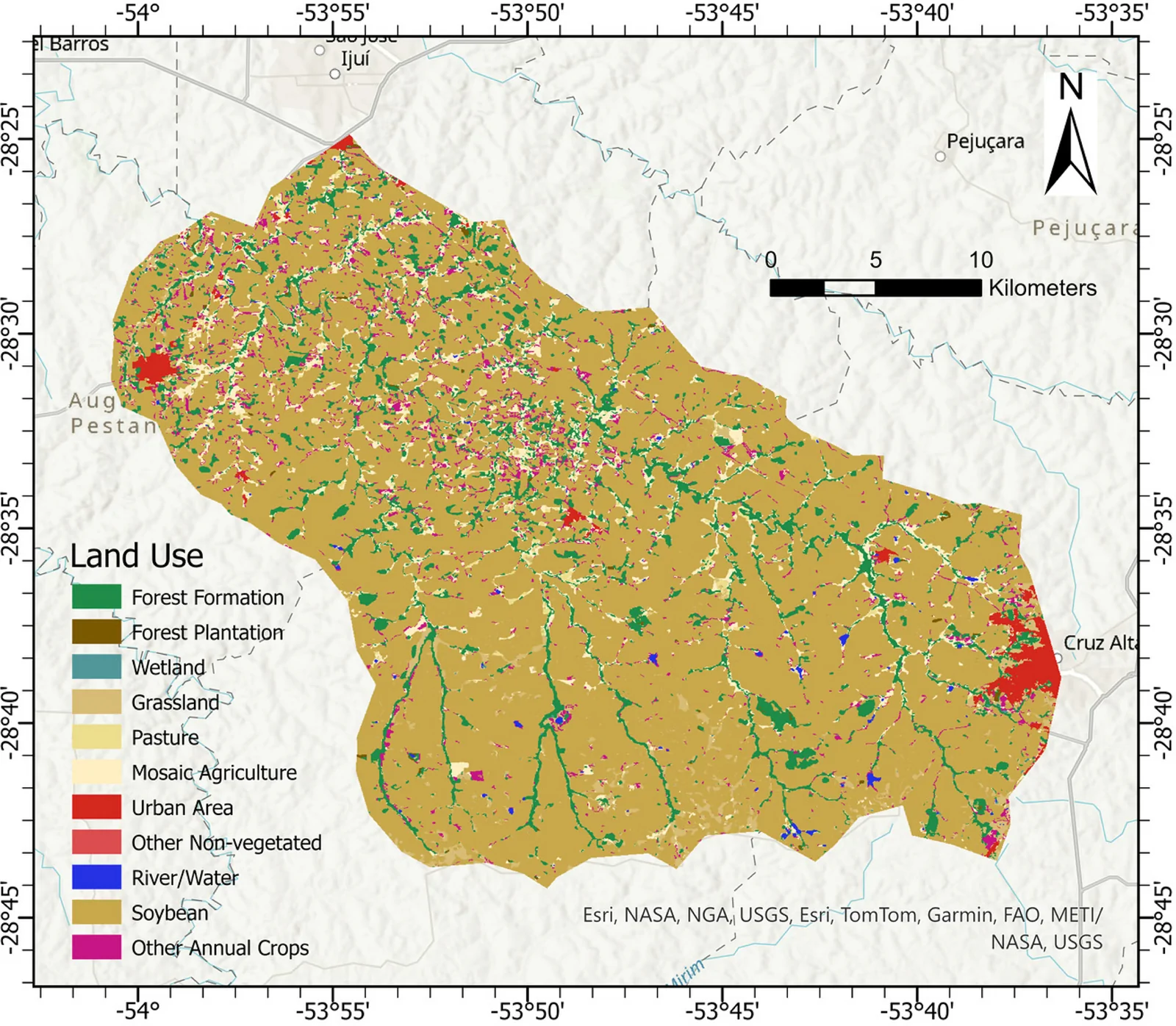
Land use map of the Conceição River catchment based on MapBiomas Collection 9 (www.MapBiomas.org)

Archived soil samples (n = 5) from Didoné et al. [28] were used to evaluate each method’s performance and reproducibility. The samples were collected in 2016 from the Conceição catchment (Fig. 1) in the state of Rio Grande do Sul, Brazil (28° 25′ 12″ S, 53° 54′ 36″ W). Intensively cultivated with soybeans, the Conceição catchment has undergone a shift in soil management practices from conventional tillage in the 1960 s to a dominance of no-tillage agriculture since the 1990s. The dominant soil type in the catchment is Ferralsol with a clay content of 45–60% [28]. The samples were collected from different layers in the upper 40 cm of the soil profile and had been previously analyzed for gamma emitter radionuclides using low-background High Purity Germanium Detectors at the *Laboratoire des Sciences du Climat et de l’Environnement* (Gif-sur-Yvette, France). The soil samples were dried at 102 °C and sieved at 2 mm. The samples used for this study were selected to cover ^137^Cs activity concentrations comprised between 0.3 and 2.9 Bq/kg to reflect the highest and lowest detectable level of Pu in the soil.

### Sample digestion and radiochemical separation

**Fig. 2:**
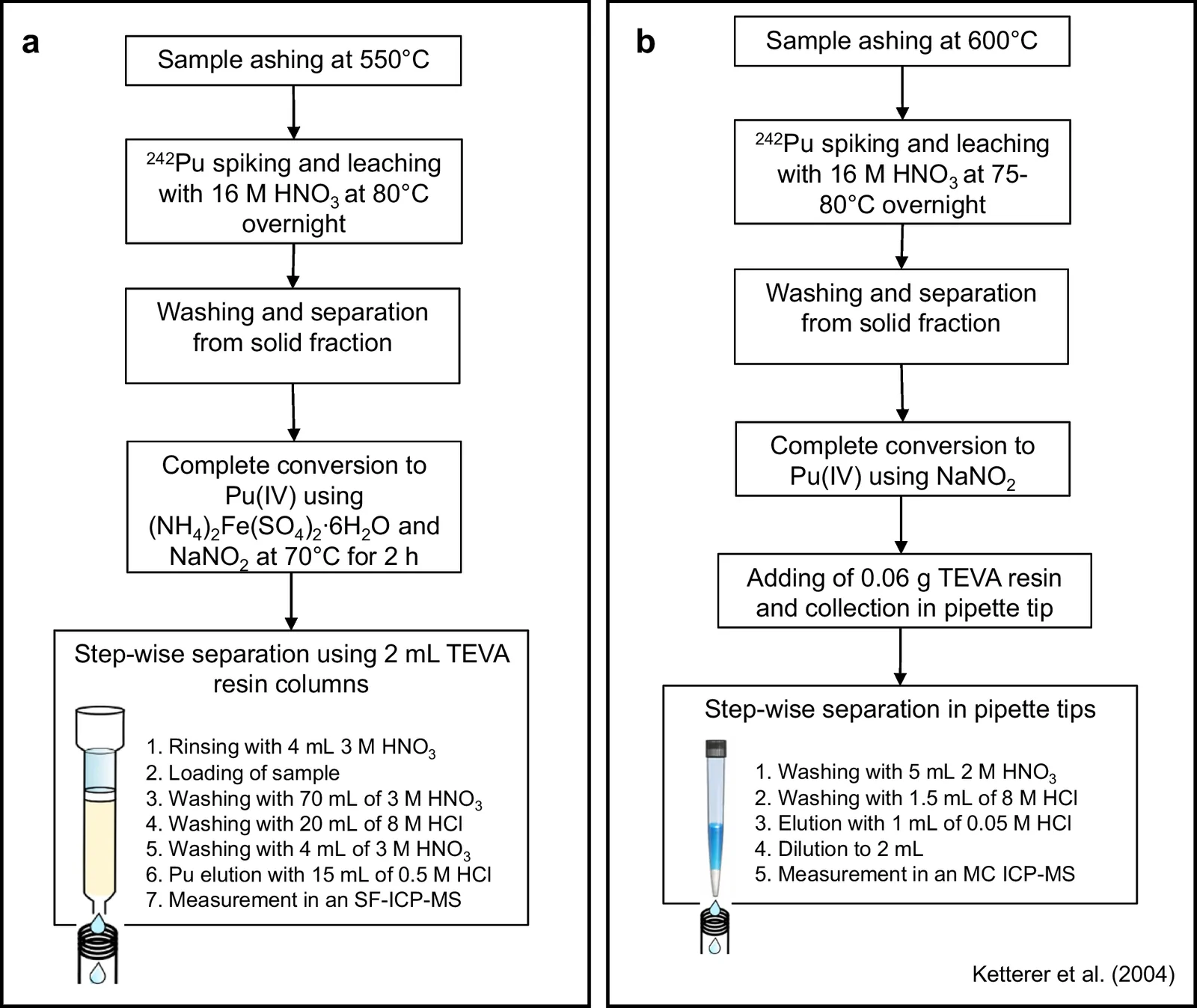
Flowcharts of the modified leaching and separation procedure of Pu isotope from soil (**a**) and the procedure proposed by Ketterer et al. [21] (**b**)

Using the leaching procedure of Ketterer et al. [21], adapted by Dowell et al. [15], we obtained low and highly variable ^242^Pu recovery rates of 45 ± 24% and ^238^U decontamination factors of 842 ± 1148 for soils from the Conceição catchment. Accordingly, most of the samples had ^239^Pu and ^240^Pu activity concentrations that were below the detection limits achieved with this method. To improve the recovery rates and separation factors, we modified the procedure of Ketterer et al. [21] (Fig. 2) and compared the performance of the methods, as detailed below.

#### Modified leaching and separation procedure (method A)

Five soil samples were dry-ashed at 550 °C for 16 h. About 5 g of the ashed soil samples was weighed into 60 mL glass vials and spiked with 1 mL of 10 pg/mL ^242^Pu standard together with reagent blanks and a standard reference material (IAEA-412). Afterwards, 10 mL of 16 M HNO_3_ was added to each vial, and they were leached in a sand bath at 80 °C for 16 h.

Following HNO_3_ leaching, 10 mL of ultrapure water was added into each vial, cooled, and transferred into 50 mL centrifuge tubes. The slurries were centrifuged at 3000 rpm for 10 min and filtered into clean 50 mL centrifuge tubes. The remaining soil in the vials was washed with 20 mL of 4.5 M HNO_3_, transferred into the centrifuge tubes with residue, centrifuged again, and filtered into the 50 mL with the first filtrates, resulting in a final concentration of 6.25 M HNO_3_.

To completely convert the Pu species in the solution to Pu(IV), 1.5 mL of 12% (NH_4_)_2_Fe(SO_4_)_2_⋅6H_2_O and 1 mL of freshly prepared 40% NaNO_2_ solutions were added sequentially into the centrifuge tubes [29]. The centrifuge tubes with the solution were then heated at 70 °C for 2 h to complete the redox process. Before separating the Pu isotopes from other actinides, each solution was allowed to cool and was diluted with 30 mL ultrapure water to reduce matrix effects, resulting in a final concentration and volume of 3.5 M HNO_3_ and 70 mL, respectively. At this concentration, Pu is selectively sorbed into the TEVA resins while U is eluted [30].

The 2 mL TEVA columns were preconditioned with 4 mL of 3 M HNO_3_, and the sample solutions were loaded into the TEVA columns with a gravitational flow rate of around 0.75 mL per min. Afterwards, the columns were washed successively with 70 mL of 3 M HNO_3_ and 20 mL of 8 M HCl to remove traces of U and Th. Before Pu elution, the columns were again washed with 4 mL of 3 M HNO_3_ to reduce the concentration of acid in the columns, and 15 mL of 0.5 M HCl was used to elute Pu from the TEVA columns.

#### Further modifications to the leaching and separation procedure (methods B-E)

We also tested the performance of other modifications to the procedure of Ketterer et al. [21] for extracting and separating Pu isotopes from the same five soil samples. For methods B–E, the 3 M HNO_3_ washing solution also contained 0.01 mM Mohr’s salt, which is known to maintain the Pu oxidation state of 4^+^ during the washing procedure and improves the elution of Pu from the TEVA column [31]. For method C, we tested whether it was possible to minimize the use of soil material to 1 g using the procedure outlined in method A. We also tested if using more acid (20 mL of 16 M HNO_3_) to leach Pu isotopes from the soil samples (method D) could improve the Pu recovery rates of the procedure (Table 1). Sample solutions were therefore diluted with more ultrapure water to reach a final concentration of ~ 3.5 M, resulting in a final volume of 120 mL which could further reduce matrix effects.

**Table 1:** Summary of different Pu extraction and separation procedures. *id.*, identical

Step	Method A	Method B	Method C	Method D	Method E*	Fusion Digestion
Sample mass	5 g	5 g	1 g	5 g	5 g	5 g
Organic matter removal	Furnaced at 550°C	id. to A	id. to A	id. to A	id. to A	id. to A
Spiking and leaching	Spiked with ^242^Pu, 10 mL 16 M HNO_3_, 80 °C for 16 h	id. to A	id. to A	spiked with ^242^Pu tracer and leached with 20 mL 16 M HNO_3_ at 80 °C for 16 h	id. to A	Spiked with ^242^Pu, fusion digestion at 1100 °C, dissolution in 4.5 M HNO_3_
Solid–liquid separation	Centrifugation, rinsing with 4.5 M HNO_3_, filtration	id. to A	id. to A	id. to A	id. to A	Precipitation of silicates with PEG and filtration
Conversion to Pu(IV)	1.5 mL 12% Mohr’s salt, 1 mL 40% NaNO_2_	id. to A	id. to A	id. to A	id. to A	3 g Mohr’s salt
Final sample concentration and volume	~ 3.5 M, 70 mL	id. to A	id. to A	~ 3.5 M, 120 mL	id. to A	4.5 M, 175 mL
TEVA column pre-conditioning	4 mL 3 M HNO_3_	id. to A	id. to A	id. to A	id. to A	10 mL 0.2% HNO_3_/0.2% HF/0.01 mM Fe(II) and conditioned with 10 mL 3 M HNO_3_/0.1 mM Fe(II)
Flow rate	~ 0.75 mL/min (gravitational)	id. to A	id. to A	id. to A	~ 2 mL/min (in vacuum box)	~ 2 mL/min (in vacuum box)
Washing/elution of other actinides	70 mL 3 M HNO_3_, 20 mL 8 M HCl, 4 mL 3 M HNO_3_	70 mL 3 M HNO_3_/0.01 mM Fe(II), 20 mL 8 M HCl, 4 mL 3 M HNO_3_/0.01 mM Fe(II)	id. to B	id. to B	70 mL 3 M HNO_3_/0.01 mM Fe(II), 30 mL 8 M HCl, 15 mL 3 M HNO_3_/0.01 mM Fe(II)	50 mL 6 M HCl and 100 mL 3 M HNO_3_/0.1 mM Fe(II)
Elution of Pu	15 mL 0.5 M HCl	id. to A	id. to A	id. to A	id. to A	20 ml 0.2% HNO_3_/0.2% HF/0.01 mM Fe(II)

*PULSE method

Finally, we tested the effect of using more acid to elute other actinides in improving recovery rates and separation factors. Specifically, we used additional 10 mL of 8 M HCl to separate Th and 15 mL of a 3 M HNO_3_/0.01 mM Fe(II) solution to improve the separation factor for U. The procedure was conducted in a vacuum box to increase the flow rate up to ~ 2 mL/min as well as the separation throughput.

#### Fusion digestion and separation procedure

The performance of the modified leaching and separation procedures (methods A–E) was compared to that of the borate fusion digestion (FD) procedure [32, 31] as routinely applied at Spiez Laboratory (Switzerland). This procedure involves spiking 5 g of sample with a known amount of ^242^Pu tracer and its melting with Spectroflux 100B (Lithium Metaborate (LiBO_2_):Lithium tetraborate (Li_2_B_4_O_7_), 80:20 w/w%), followed by silicates removal by precipitation with Polyethylene glycol (PEG) and subsequent Pu separation on TEVA columns in a vacuum box (Table 1). The TEVA columns were pre-conditioned with 10 mL of a 0.2% HNO_3_/0.2% HF/0.01 mM Fe(II) solution and then with 10 mL of a 3 M HNO_3_/0.1 mM Fe(II) solution. After loading the samples, the TEVA columns were rinsed with 50 mL of 6 M HCl and then with 50 mL of a 3 M HNO_3_/0.1 mM Fe(II) solution. This technique is expected to release all Pu from the soil, even if there are hot particles containing Pu (refractory particles), and reduce matrix effects. The method has been reported to achieve high recovery rates of 80 (± 13) % and low detection limits of 0.002 and 0.008 Bq/kg for ^239^Pu and ^240^Pu, respectively [31, 32]. The method FD had also been validated previously with a range of certified reference materials (Table 2), which makes it a suitable standard method against which the optimized leaching and separation procedures may be reliably tested.

**Table 2:** Measured ^239^Pu and ^240^Pu values in certified reference materials using the method FD

Certified reference material	Certified values		Measured values		Reference
	^239+240^Pu (Bq/kg)	^240^Pu/^239^Pu (atom ratio)	^239+240^Pu (Bq/kg)	^240^Pu/^239^Pu (atom ratio)	
IAEA-375	0.30 ± 0.04	0.27 ± 0.01	0.24 ± 0.02	0.31 ± 0.05	[31]
IAEA-326	0.49 ± 0.02	0.19 ± 0.01	0.50 ± 0.03	0.18 ± 0.02	
IAEA-327	0.58 ± 0.02	0.19 ± 0.02	0.59 ± 0.03	0.19 ± 0.02	
IAEA-447	5.3 ± 0.2	0.19 ± 0.01	5.34 ± 0.13	0.18 ± 0.01	
IAEA-368	31.50 ± 2.50	0.04 ± 0.01	34.0 ± 0.9	0.032 ± 0.001	
IAEA-367	37.10 ± 2.70	0.296 ± 0.008	37.7 ± 0.8	0.30 ± 0.01	
IAEA-384	106.50 ± 3.50	0.05 ± 0.01	107 ± 3	0.050 ± 0.001	
IAEA-326	0.50 ± 0.02	0.19 ± 0.01	0.48 ± 0.02	0.19 ± 0.01	[32]
IAEA-327	0.58 ± 0.02	0.19 ± 0.02	0.61 ± 0.03	0.19 ± 0.02	
IAEA-soil6	1.04 ± 0.08	0.187 ± 0.004	1.08 ± 0.03	0.186 ± 0.007	
IAEA-135	213 ± 11	0.204 ± 0.005	211 ± 5	0.204 ± 0.005	
IAEA-300	3.55 ± 0.06	0.183 ± 0.007	3.62 ± 0.09	0.183 ± 0.007	

### Pu analysis by SF-ICP-MS

Aqueous solutions containing the Pu fractions were introduced into the SF-ICP-MS with a SC-2 autosampler, an Apex nebulizing system connected to an ACM desolvator, and a self-aspirating PFA-ST-nebulizer (all from Elemental Scientific Incorporation, Omaha, USA). All measurements were performed in the low-resolution mode (m/Δm = 300). In this configuration, the sensitivity for U was about 9 × 10^6^ s^−1^ per ng/mL ^238^U compared to a sensitivity of about 1 × 10^6^ s^−1^ per ng/mL ^238^U with the advanced spray chamber (Finnigan). The electronic background signal was measured on mass 220 amu and was less than 0.2 s^−1^. Oxide rates for UO and CeO were lower than 0.3%. An uptake time of 1 min was enough to get stable signals. The total acquisition time per sample was 5 min. U and Th interferences due to hydride generation and tailing were corrected mathematically. The interference correction factors were determined with 20 ng/mL U and 100 ng/mL Th standard solutions. Pu isotope concentrations were calculated from the signal of the ^242^Pu tracer.

### Post-processing and calculations

The plutonium isotope signals were first corrected for the interferences from U and Th and for the blank measured after every 6–8 samples. The plutonium isotope concentrations in the soil sample were calculated from the response factor of the ^242^Pu tracer. The response factor was calculated as the quotient of the net signal intensity of ^242^Pu and the concentration of the ^242^Pu in the soil due to the addition of the ^242^Pu tracer. The same response factor was applied to all plutonium isotopes because the ICP-MS sensitivity is essentially constant across masses 237–244. 

The activity concentration of the plutonium isotopes in soil was calculated by multiplying the isotope concentrations with the specific activities,

$${A}_{Pu-i,soil}={c}_{Pu-i,soil}\times {SA}_{Pu-i}\times {10}^{-15}$$

where ${A}_{Pu-i,soil}$ is the activity concentration of the plutonium isotope i in soil in Bq/kg, ${c}_{Pu-i,soil}$ is the plutonium isotope concentration in dry soil in fg/g, and ${SA}_{Pu-i}$ is the specific activity of the plutonium isotope in Bq/kg. The specific activities used were 2.298 × 10^12^ Bq/kg for ^239^Pu and 8.407 × 10^12^ Bq/kg for ^240^Pu.

Chemical recovery was determined based on the amount of ^242^Pu tracer added and the amount of ^242^Pu the eluate. The ^242^Pu concentration in the eluate was determined with a one-point external calibration. Indium was used as an internal standard.

$${Y}_{Pu-242}=\frac{{I}_{Pu-242,net}}{Res{p}_{Pu,eluate}}\times \frac{{v}_{eluate}}{{v}_{spike}}\times \frac{1}{{c}_{Pu-242,spike}}\times 100\%,$$

where ${Y}_{Pu-242}$ is the tracer recovery in percent, ${I}_{Pu-242,net}$ is the net signal intensity of ^242^Pu in cps after blank correction, $Res{p}_{Pu,eluate}$ is the instrument response factor in cps per ppq in the eluate, ${v}_{spike}$ is the mass of spike added in mL, ${v}_{eluate}$ is the total eluate volume in mL, and ${c}_{Pu-242,spike}$ is the certified concentration of the 242Pu spike in ppq.

^238^U separation factors were calculated as the quotient of the ^238^U to ^242^Pu recovery rates after separation on TEVA columns. The initial ^238^U concentrations were obtained by measuring the samples by gamma spectrometry prior to leaching, or by analyzing aliquots of the solutions resulting from fusion digestion by ICP-MS. It should be noted that for the modified leaching procedures (methods A–E), partial reduction in total ^238^U concentration already occurs due to the non-leaching of sillicate-bound U. Consequently, the separation factors of leaching procedures reflect the combined effect of incomplete U extraction during leaching and U removal on the TEVA column. On the other hand, fusion digestion (method FD) dissolves all matrix phases including all U in the soil, so the SF reflects only the TEVA separation performance. Thus, the separation factors of the modified leaching and fusion digestion procedures are not directly equivalent, although both represent the net U reduction achieved relative to Pu in the final solution after the TEVA separation.

Interference corrections due to hydride formation and tailing of ^238^U, ^232^Th, and contamination of the ^242^Pu tracer with other Pu isotopes were calculated and subtracted from the measured signal to obtain the net analyte signal. ^238^U and ^232^Th contributions were calculated using tailing ratios measured on pure element calibration standards. The concentrations of the minor Pu isotopes in the ^242^Pu tracer from the tracer certificate were used to calculate the contributions due to the ^242^Pu tracer added. The total interference on mass m-i was calculated as:

$${I}_{interf,m-i}=\left({R}_{\frac{U}{m}-i}\times {I}_{U238}\right)+\left({R}_{\frac{Th}{m}-i}\times {I}_{Th232}\right)+\left({R}_{\frac{Pu}{m}-i}\times {I}_{Pu-242}\right)+{I}_{blank},$$

and the corrected analyte signal was then determined as:

$${I}_{net,m-i}={I}_{sample,m-i}-{I}_{interf,m-i},$$

where ${I}_{interf,m-i}$ is the calculated interference signal at mass m-i in cps, obtained from the sum of the tailing contributions from matrix elements and the reagent blank. The terms ${R}_{U/m-i}$, ${R}_{Th/m-i}$, and ${R}_{Pu/m-i}$ represent the tailing ratios of uranium, thorium, and plutonium at mass m-i. The variables ${I}_{U238}$, ${I}_{Th232}$, and ${I}_{Pu242}$ are the signal intensities of ^238^U, ^232^Th, and ^242^Pu in cps. ${I}_{blank}$ is the blank signal in cps, ${I}_{sample,m}$ is the measured sample signal at mass m in cps, and ${I}_{net,m}$ is the final analyte signal after blank and interference correction in cps.

The detection limit (DL_Pu-i_) for a plutonium isotope i was calculated using the three-sigma criterion based on the standard deviation of the interference correction for the mass i and propagated through the same calculation chain as the measured activity:

$${DL}_{Pu-i}=\frac{3\times {\sigma}_{interf,m-i}}{Res{p}_{Pu,soil}}\times {SA}_{Pu-i}\times {10}^{-15}$$

where ${\sigma}_{interf,m-i}$ is the standard deviation of the interference contribution in cps, $Res{p}_{Pusoil}$ is the response factor in cps per ppq (dry soil), and $S{A}_{Pu}$ is the specific activity of the isotope i in Bq/kg.

The expanded or total analytical uncertainty (k = 2) was calculated following the ISO Guide to the Expression of Uncertainty in Measurement using the root-sum-of-squares method:

$${U}_{c-i}=2\sqrt{{u}_{m,sample}^{2}+{u}_{m,spike}^{2}+{u}_{mass discr}^{2}+{u}_{{I}_{net,Pu-242}}^{2}+{u}_{{I}_{net,Pu-i}}^{2}},$$

where ${U}_{c-i}$ is the expanded combined uncertainty expressed in percent at a coverage factor of two corresponding to approximately 95% confidence for isotope i, ${u}_{m,sample}$ is the uncertainty of the sample mass (dry mass), ${u}_{m,spike}$ is the uncertainty of the mass of the ^242^Pu tracer added, ${u}_{mass discr}$ is the uncertainty of the mass discrimination, ${u}_{{I}_{net,Pu-242}}$ is the uncertainty of the interference corrected ^242^Pu signal, and ${u}_{{I}_{net,Pu-i}}$ is the uncertainty of the interference corrected Pu-i signal.

### Statistical analyses and application of the optimal procedure

ANOVA was used to determine significant differences between methods in terms of recovery rates and ^238^U separation factors. Data for ^238^U separation factors were exponentially transformed to achieve normal distribution. To find out which specific methods are different, the Tukey’s HSD post-hoc test was performed. All statistical analyses were carried out in R [33].

Finally, the optimal leaching and separation procedure was used to determine the ^239^Pu and ^240^Pu of soil samples (n = 218) collected from different soil layers all over the Southern Hemisphere, representing mainly Oceania and South America. This set of samples thus covers a wide range of ^239,240^Pu and ^238^U contents and sources, as well as soil types.

## Results and discussion

### Pu isotope activities and isotopic ratios

**Fig. 3:**
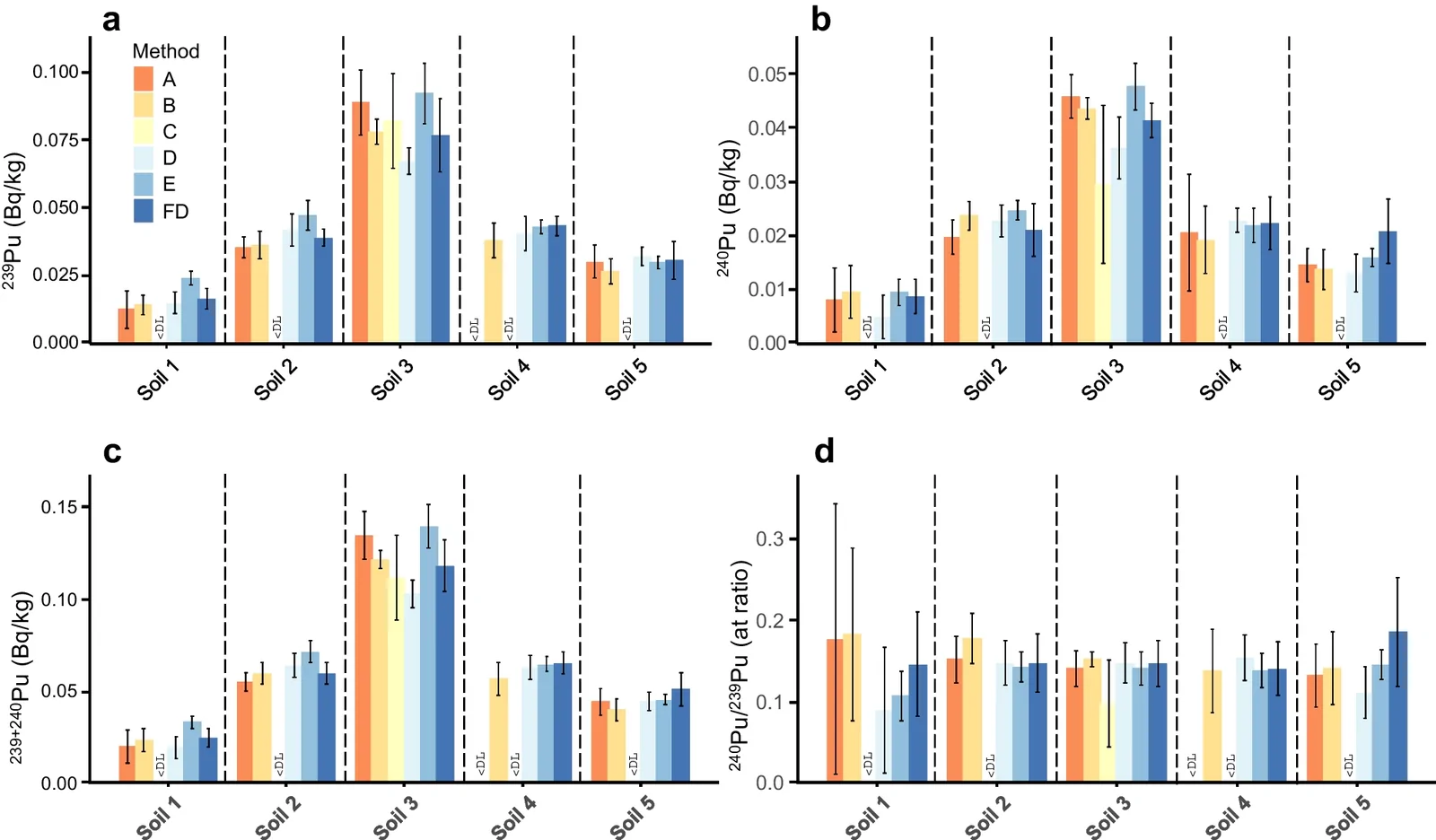
Activity values measured for ^239^Pu and ^240^Pu isotopes (**a**, **b**), ^239+240^Pu (**c**), and ^240^Pu/^239^Pu atomic ratios (**d**) for tested soils. The error bars show expanded uncertainties (k = 2). Method E is the PULSE method

The measured ^239^Pu and ^240^Pu (and its sum^239+240^Pu) activities were generally consistent across the different methods (Fig. 3a–c), with Soil 3 having the highest (^239+240^Pu: 0.124 ± 0.014 Bq/kg) and Soil 1 the lowest (^239+240^Pu: 0.024 ± 0.006 Bq/kg) activity values (see Table S1). However, following method C (e.g., standard method A but only 1 g of soil), the use of only 1 g of soil was not sufficient, resulting in ^239^Pu, ^240^Pu, and ^239+240^Pu activities that were mostly below the detection limit.

In general, the results indicate that the values obtained using methods A–E and the fusion digestion procedures agree within analytical uncertainty. This also implies that acid-leaching efficiently extracted the fallout-associated Pu fraction in these Ferralsols. It is also important to note that the dry-ashing temperature is a critical factor that affects the leaching of Pu from soils. Dry-ashing above 600 °C may reduce the amount of leachable Pu by 40% through the formation of refractory materials, which are insoluble in HNO_3_ [34]. This is consistent with the considerable reduction recoveries observed in our ashing experiments previously (see Table S2), justifying our choice to ash the soil samples at 550 °C. In addition, keeping the temperature at 550 °C also prevents the destruction of volatile salts and inorganic compounds in the soil [35].

Similarly, the ^240^Pu/^239^Pu atomic ratios were in general consistent across methods for most of the soils (Fig. 3d), with a mean value of 0.145 ± 0.022. This is also consistent with the ^240^Pu/^239^Pu atomic ratio of 0.14 ± 0.01 previously reported for soils collected from the same catchment and further confirms that radioactive fallout from French nuclear tests in Polynesia may have contributed roughly 30% to the total Pu inventory in this region [7]. The ^240^Pu/^239^Pu atomic ratios were also consistent across methods, except for method C, which only obtained detectable Pu isotope values for Soil 3. This indicates that ^240^Pu/^239^Pu atomic ratios are method-independent and that no isotopic fractionation occurred during chemical extraction and separation using the modified leaching and separation procedures. However, it has to be noted that low Pu content in the samples could lead to significant uncertainties in the ^240^Pu/^239^Pu atomic ratios. This should therefore be incorporated in error propagation in unmixing models which intend to quantify the contribution of different Pu fallout sources in soils and sediments [4, 7].

### ^242^Pu recovery rates and ^238^U separation factors

**Fig. 4:**
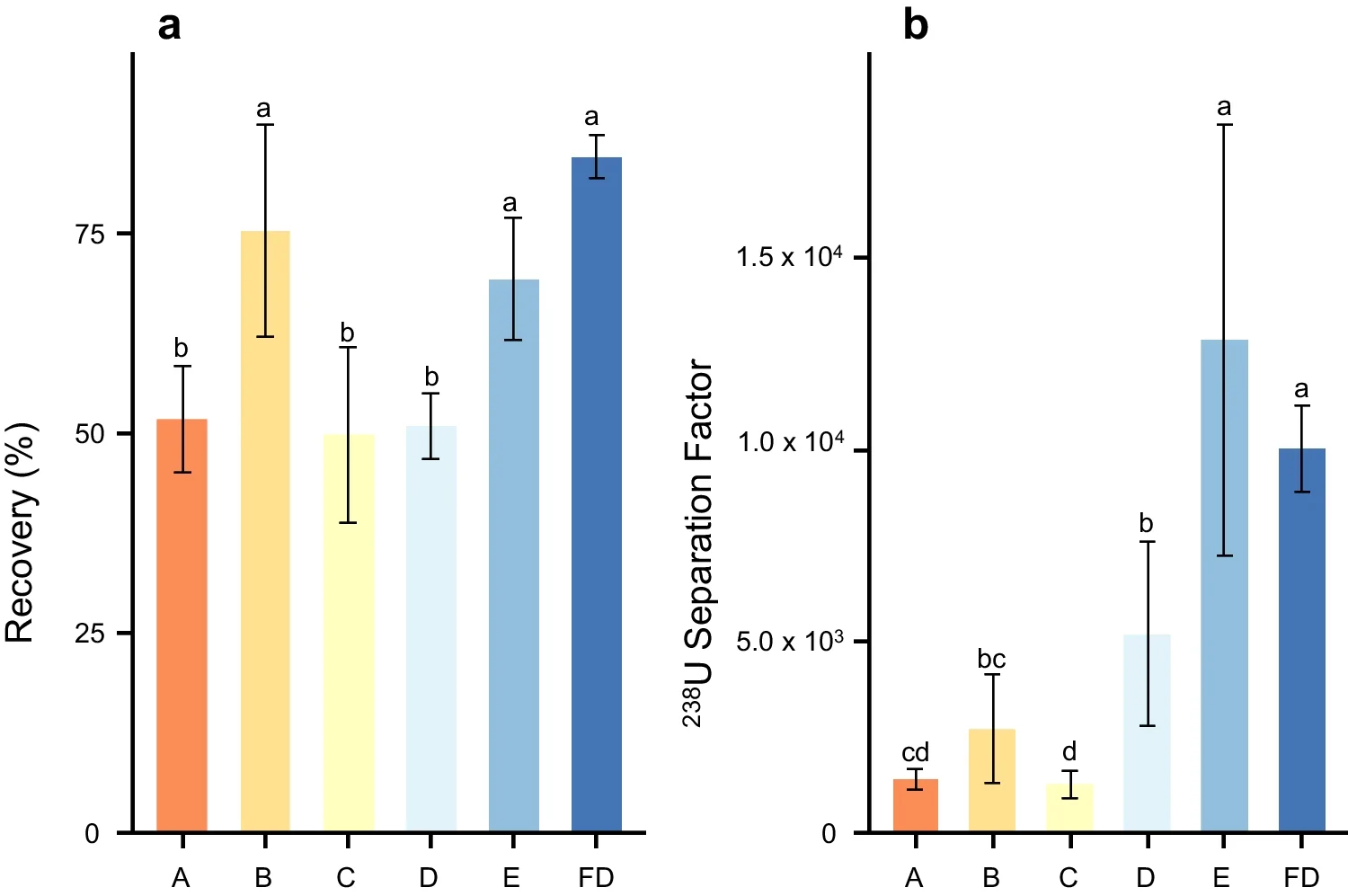
Means of ^242^Pu spike recovery percentages (**a**) and ^238^U separation factors (**b**) for the different modified Ketterer procedures (Methods A–E) and fusion digestion procedure (Method FD). Methods that are not sharing the same letters above each bar were significantly different at *p* < 0.05. Method E is the PULSE method

On average, the fusion digestion procedure yielded a recovery rate of 84 (± 3) % (Fig. 4a, Table S3), which is consistent with the values reported by Sahli et al. [31]. Among the modified leaching and separation procedures, methods B obtained the highest recovery among the leaching procedures (75 ± 13%), followed by Method E (69 ± 8%). The rest of the procedures yielded a recovery rate of mostly over 50%. Interestingly, the ^242^Pu recovery of method D, which used 20 mL of concentrated acid for leaching (instead of 10 mL), is one of the lowest (50 ± 4%). This indicates that increasing the acid volume for leaching did not improve the recovery. This corroborated the findings of Ketter et al. [21] and Dowell et al. [25] showing that a 1:2 soil-to-acid ratio for leaching is sufficient to leach majority of the matrix-associated Pu.

In terms of ^238^U separation factors, method E obtained the highest value of 13 (± 6) × 103, which was higher but not significantly different from the fusion digestion procedure’s separation factor of 10 (± 1) × 10^3^. Methods A–D obtained lower separation factors between 1 and 10 × 10^3^, which were significantly lower than those obtained for method FD (Fig. 4b). Only methods E and FD have high mean separation factors on the order of at least 10^4^, which is generally sufficient for accurate Pu analysis in samples not containing high U content [16, 31]. It should be noted, however, that the ^238^U content prior to TEVA separation may not be the same between the modified leaching and fusion digestion procedures (see).

High ^242^Pu recovery rates and ^238^U separation factors are considered the benchmarks of a robust Pu analytical method. This is achieved by effectively extracting Pu from soil and sediment samples and separating it from ^238^U and other actinides, which form interferences during the measurement in ICP-MS. Table 2 summarizes the different extraction and separation procedures used for measuring ultra-trace Pu isotopes in ICP-MS from the literature. Aside from the extraction procedures tested in this study, other commonly used procedures include HNO_3_-HF and aqua regia leaching, with both reportedly resulting in 50–90% tracer recovery [20, 23, 36] similar to the methods tested in this study (Table 3). For ^238^U separation, different resins are also used such as AG 1-×4 anion exchange AG MP-1 M aside from TEVA. Coprecipitation is sometimes employed to improve ^238^U separation factors [20, 36, 37], which were found to be one to three orders of magnitude greater than those achieved in the tested procedures (Fig. 3, Table 3). However, as it requires a series of additional steps needing additional pH adjustments, reagents, and solid-liquid separation, coprecipitation is not convenient for routine analysis of a large number of samples. For separation procedures for later measurement in an ICP-MS/MS, residual ^238^U after the chemical separation step is addressed within the instrument during analysis [25, 36].

**Table 3:** Method performance of different ultra-trace level Pu isotope extraction and separation procedures from the literature in comparison to this study

Instrument	Sample mass	Sample source	Pu extraction and separation procedure	^239+240^Pu (Bq/kg)	^242^Pu recovery (%)	^238^U separation factor	Reference
Elemental SF-ICP-MS	5 g	Switzerland	Fusion digestion, TEVA columns	~ 0.3–1.5	50–80	2 × 10^4^	[32]
Thermo Fisher Scientific X Series II ICP-MS	30–60 g	Denmark and China	Aqua regia leaching, coprecipitation, AG 1-×4 anion exchange combined with TEVA columns	0.475—0.966	52 ± 12	3.8 × 10^5^	[23]
Elemental SF-ICP-MS	5 g	Switzerland	Fusion digestion, TEVA columns	0.02–2.63	80 ± 13	1 × 10^4^	[31]
Agilent 8800 ICP-MS/MS	10–20 g	Eastern China	Aqua regia leaching, coprecipitation, AG 1-×4 anion exchange and TEVA columns	0.011—0.27	80–90	N/A	[36]
Thermo Fisher Scientific Element XR SF-ICP-MS	2 g	Japan	HNO_3_-HF leaching, coprecipitation, AG MP-1 M resin	0.192—0.857	72 ± 13	1.8 × 10^6^ - 1.7 × 10^7^	[20]
Agilent 8900 ICP-MS/MS	50 g	Oroba Valley, Kenya	HNO_3_ leaching, TEVA columns	0.09 ± 0.003	81 ± 15	200	[25]
Elemental SF-ICP-MS	5 g	Southern Brazil	HNO_3_ leaching, TEVA columns	0.020—0.135	50 ± 11 - 75 ± 13	1.3 × 10^3^ - 1.3 × 10^4^	This study (methods A–E)
Elemental SF-ICP-MS	5 g	Southern Brazil	Fusion digestion, TEVA columns	0.020—0.135	84 ± 3	1.0 × 10^4^	This study (method FD)

Despite using a minimal amount of sample and relatively simple procedures, the values that we have obtained are comparable with the recovery rates and separation factors reported in the literature (Table 3). Among the modified leaching and separation procedures tested in this study, method E achieved a lower recovery rate than FD (69 ± 8% vs. 84 ± 3%), but a higher mean ^238^U SF (1.29 × 104 vs. 1.00 × 104). These results indicate that HNO_3_ leaching is sufficient to achieve acceptable recovery rates comparable to the values reported in the literature, and even a low sample mass does not require a coprecipitation step to achieve high ^238^U separation factors.

### Uncertainties and detection limits

Measurement uncertainties and detection limits across the methods tested in this study are generally in the mBq/kg level (Table 4). Methods B and D had uncertainties and detection limits that were comparable to the values obtained by the fusion digestion procedure (method FD). Among the methods, E achieved the lowest levels of uncertainty and detection limits, surpassing even those of the fusion digestion procedure (FD). Notably, these values were also lower than those of method D, despite the latter involving greater dilution of the leachates to reach a ~ 3.5 M HNO_3_ concentration in the final sample solution. This indicates that increased dilution alone did not improve analytical precision or detection capability and that separation in the TEVA resin column appeared to be a more critical factor controlling measurement performance.

**Table 4:** Mean (± SD) of measurement uncertainties (k = 2) and detection limits of Pu isotopes across methods

Method	^239^Pu uncertainty (mBq/kg)	^239^Pu DL (mBq/kg)	^240^Pu uncertainty (mBq/kg)	^240^Pu DL (mBq/kg)	^239+240^Pu uncertainty (mBq/kg)	^239+240^Pu uncertainty (%)	^240^Pu/^239^Pu uncertainty (atomic ratio)
A	7.24 (3.53)	12.11 (13.15)	5.42 (3.2)	5.08 (4.95)	8.5 (3.42)	19.63 (16.59)	0.06 (0.07)
B	4.89 (1.02)	5.8 (2.82)	3.92 (1.75)	2.21 (1.44)	6.4 (1.52)	14.01 (7.82)	0.05 (0.04)
C^*^	17.59	28.30	14.69	26.84	22.91	20.53	0.05
D	4.96 (1.22)	6.66 (2.09)	3.66 (1.31)	2.47 (0.67)	6.31 (0.99)	13.64 (8.7)	0.04 (0.02)
E**	4.8 (3.79)	2.74 (1.3)	2.7 (1.08)	0.83 (0.64)	5.64 (3.69)	7.96 (1.86)	0.02 (0.005)
FD	6.23 (4.35)	8.29 (0.45)	4.43 (1.24)	5.89 (0.27)	8.01 (3.65)	13.75 (4.88)	0.05 (0.02)

*Single values were provided for method C because all other samples were below detection limit. **PULSE method

Detection limits and uncertainties are key criteria for evaluating the suitability of a Pu extraction and separation procedure for a given Pu concentration in the soil. For determining ultra-trace-level Pu activity concentrations, analytical limits need to be sufficiently low for obtaining reliable results. The mBq/kg level uncertainties and detection limits for both isotopes with the tested methods were close to the values previously reported [15, 20, 23], with an uncertainty range of 0.0065–0.0168 Bq/kg and detection limits of 0.0004–0.003 Bq/kg. This corresponds to a ^239+240^Pu uncertainty range of 1.9 ± 0.4%–13.1 ± 5.8% [15, 20, 23, 36]. Methods B, D, and E therefore compare well with these values despite not using an ICP-MS/MS nor employing a coprecipitation step.

### Selecting the optimal leaching and separation procedure

By comparing the isotopic values, recovery rates, separation factors, uncertainties, and detection limits, we determined which method is the most suitable for conducting ultra-trace-level Pu measurements in soils. Method C used insufficient mass, which resulted in below detection limit values upon measurement, while method D used high acid volumes before separation without improving recovery and analytical limits. Method A also had the lowest recoveries and separation factors along with methods C and D, and we thus recommend to likewise exclude them. Although Method B achieved a higher recovery rate (75 ± 13%) than Method E (69 ± 8%), Method E obtained a substantially higher ^238^U separation factor (1.3 × 10^4^ vs. 2.7 × 103) and lower detection limits (^239^Pu: 2.7 vs. 5.8 mBq/kg; ^240^Pu: 0.83 vs. 2.2 mBq/kg).

The trade-off between ^242^Pu tracer recovery and ^238^U separation efficiency is a critical consideration in ultra-trace Pu analysis by sf-ICP-MS, where residual ^238^U causes isobaric interference on ^238^Pu and spectral tailing onto ^239^Pu, propagating directly into measurement uncertainty and detection limits. In isotope dilution, the loss of recovery is usually corrected by the 242Pu tracer, resulting in still accurate values as previously reported [23, 31] despite losing up to over 50% of the tracer. On the other hand, insufficient ^238^U removal or low SFs (Methods A–B; Fig. 4b) lead to higher uncertainties and detection limits (Table 4). Method E achieved the optimal balance, with a mean recovery of 69 ± 8% and a mean SF of 1.3 × 10^4^, which is approximately 2.5-fold higher than Method D (5.2 × 10^3^) and 5–10 times higher than Methods A–C. The superior SF of Method E resulted in the lowest uncertainties and detection limits, making it the most reliable procedure for ultra-trace Pu measurements. The use of additional 10 mL of 8 M HCl and 15 mL of 3 M HNO_3_/0.01 mM Fe(II) during the separation step proved critical in improving the analytical limits of the procedure, while the use of the vacuum box to increase the flow rate did not affect its overall performance.

To make it easy for future reference, we refer to method E as the “PULSE method” (Plutonium extraction from Soils at Low concentrations with high prEcision). Based on the analysis of 218 samples (Table 5), we have demonstrated that the PULSE method (Fig. S1) is also applicable to a wide range of ^239,240^Pu and ^238^U contents and soil types besides the Brazilian Ferralsol samples used in testing the methods.

**Table 5:** Analytical performance summary of the PULSE method as applied in measuring ^239^Pu and ^240^Pu of samples collected from different areas in the Southern Hemisphere (n = 218)

Parameter	Mean	SD	5th percentile	95th percentile
^242^Pu tracer recovery (%)	72.7	10	54.5	86.9
^238^U separation factor (n = 196)ᵃ	3.00 × 10^4^	3.05 × 10^4^	8.79 × 10^3^	5.93 × 10^4^
^239^Pu detection limit (mBq/kg)	1.81	1.34	0.68	4.18
^240^Pu detection limit (mBq/kg)	1.74	1.11	0.43	3.83
^239^⁺^240^Pu activity (Bq/kg) (n = 203)ᵇ	0.0755	0.0724	0.0073	0.1867
^239^⁺^240^Pu activity uncertainty (%)	8.6	6.3	4	23.4
^240^Pu/^239^Pu atomic ratio (n = 203)ᵇ	0.147	0.035	0.11	0.216
^240^Pu/^239^Pu ratio uncertainty	0.028	0.031	0.008	0.079

^a^Separation factor n = 196 (only for samples with known U content)

ᵇ15 samples < DL; statistics on detected values only. Uncertainties expanded (k = 2, ~ 95% confidence), ISO GUM. Detection limits: 3-sigma criterion

### Limitations

The TEVA resin used in this study was pre-packed 2 mL columns with 100–150 µm particle size. Thus, the specifications such as the acid volume for washing in the modified leaching and separation procedures may only be applicable to this type of TEVA column. It has been shown that different column and particle sizes influence the recovery of Pu from the sample [22]. As such, acid volumes for washing may require adjustment, although we expect the overall reliability of the procedure to be unaffected.

The type of soil selected in this study reflects the low level of Pu that can be expected in the Southern Hemisphere [4, 38, 39]. However, the method comparisons were only done for a specific soil type, which is Ferralsol [28], characterized by a high clay fraction content (> 60%) composed of kaolinite and abundant Fe oxides such as goethite and hematite [40]. While the clay content is high, kaolinite has a comparatively low internal surface area and lower sorption capacity than, for example, illite. Consistent with this, 13⁷Cs sorption is higher in intermediate-weathered temperate soils dominated by illite, but lower in highly weathered soils dominated by kaolinite and in young volcanic soils containing allophane and mica [41]. While the PULSE method has shown wider applicability for samples collected from all over the Southern Hemisphere (Table 5), it is not known how the other leaching and separation procedures will perform with the 218 samples collected from these other areas in the Southern Hemisphere.

Method testing was also only done on bulk soil; thus, it is not known how method performance will vary across different particle sizes, organic matter fractions, and mineralogies within the soil. As previously observed with ^137^Cs, high radiocesium interception potential or ^137^Cs adsorption was reported for intermediate-weathered soils with high content of illite [41]. However, Ferralsols are also rich in Fe and Al oxides (e.g., goethite, hematite, and gibbsite), which provide highly reactive sorption sites and can significantly enhance Pu retention via surface complexation [12, 27]. For Pu, sorption is also dependent on redox potential and pH [42], as well as biorelevant chelators and metalloproteins [43]. In addition, the effective charge of the Pu cation, which varies in the environment, also dictates the type and strength of its binding mechanism with humic substances and mineral surfaces [44]. All these will inadvertently affect the efficiency of the extraction and separation procedure, as evidenced by the variable analytical performance across different soils (Table 5).

The PULSE method effectively extracted the fallout-associated Pu fraction in the soil. However, this may not be enough when Pu is present as “hot” particles, formed through high-temperature events that lock it into robust crystalline oxides, carbides, and alloys shielded in metallic matrices [45, 46]. In this case, the use of HF-assisted digestion, borate fusion, or other type of total dissolution procedure might be more appropriate [47].

At present, there is no certified reference material available with ultra-low-activity concentrations of ^239^Pu and ^240^Pu similar to that found in the soil samples analyzed in this study. We attempted to cross-check method performance using the IAEA-412 reference material (Pacific Ocean sediment), and the PULSE method successfully reproduced the reported ^239^Pu and ^240^Pu values (Table S4). However, this method validation remains limited, because the sediment matrix differs substantially from that of soils, and its much higher Pu activity levels are not directly comparable to the ultra-low activities found in our samples. In addition, independent validation using other spectrometric techniques such as ICP-MS/MS could also be done to confirm the method performance observed in this study.

## Conclusions

^239^Pu and ^240^Pu isotopes are increasingly being recognized as a robust long-term alternative to other radionuclides for tracing geomorphological processes such as soil redistribution assessments, contamination source apportionments, and lake core sediment dating. However, most methods were optimized for measuring Pu in samples with relatively high activity concentrations (> 0.1 Bq/kg) using a relatively large sample mass (~ 10–50 g), while employing complex procedures and more sensitive instruments. Thus, for a global application, it is crucial to optimize a practicable and scalable method for low-activity soils such as those found in equatorial regions and in the Southern Hemisphere, where fallout is about four times lower than in the Northern Hemisphere. Furthermore, for lake sediment dating, methods are needed for ultra-low sample weights (< 1 g).

Among the modified leaching and separation procedures that we evaluated, the PULSE method stood out as the optimal method whose recovery is lower but within the acceptable range for the fusion digestion procedure (69 ± 8% vs. 84 ± 3%) and the one with the highest ^238^U separation factor. This allowed for more consistent results with lower uncertainties and detection limits in the range of detection limits below 3 mBq/kg for ^239^Pu and below 1 mBq/kg for ^240^Pu. All these were achieved while keeping the acid volume to the minimum and the method relatively simple and straightforward, without sacrificing performance. The PULSE method reduced reagent consumption and higher sample throughput, which reduces the total burden in terms of workload and reagent use of ultra-trace Pu analysis.

## Supplementary Information

Below is the link to the electronic supplementary material.

Supplementary file1 (PDF 183 KB)

## Data Availability

All data supporting the findings of this study are included in this article and its supplementary information.
