# Supplementary material for: Ultra-trace 239Pu and 240Pu determination in soils using optimized leaching, vacuum-assisted TEVA separation, and isotope dilution ICP-MS
Source: Anal Bioanal Chem. 2026 Jun 12;418(17):5443–57. doi: 10.1007/s00216-026-06614-y (PMC13652913; doi:10.1007/s00216-026-06614-y)
Supplement: Supplementary file 1 [file 216_2026_6614_MOESM1_ESM.pdf]

Supplementary Material for

Ultra-trace ^{239}Pu and ^{240}Pu determination in soils using optimized leaching, vacuum-assisted TEVA separation, and isotope dilution ICP-MS

Gerald Dicen[1], Floriane Guillevic[1], Judith Kobler[1], José A. Corcho Alvarado[2], Stefan Röllin[2], Olivier Evrard[3], Christine Alewell[1]

[1]Environmental Geosciences, Department of Environmental Science, University of Basel, Bernoullistrasse 30, 4056 Basel, Switzerland

[2]Spiez Laboratory, Federal Office for Civil Protection, Austrasse, 3700 Spiez, Switzerland

[3]Laboratoire des Sciences du Climat et de l'Environnement (LSCE/IPSL), Unité Mixte de Recherche 8212 (CEA-CNRS-UVSQ), Université Paris-Saclay, F-91191 Gif-sur-Yvette, France

Correspondence to: Gerald Dicen (gerald.dicen@unibas.ch)

ORCID: https://orcid.org/0000-0002-3759-9825

Incl. Tables S1-S4 and Figure S1

Table S1. Results of the ICP-MS measurement of different soil samples extracted and separated using the tested procedures. Method E is the PULSE method described in the main article.

Sample Name	Soil	Method	Recovery (%)	^{238}U SF	$^{239+240}$Pu (Bq/kg)	Unc (Bq/kg)	Unc (%)	^{239}Pu (Bq/kg)	Unc (Bq/kg)	Unc (%)	DL	^{240}Pu (Bq/kg)	Unc (Bq/kg)	Unc (%)	DL	^{240}Pu/^{239}Pu (atom ratio)	Unc (%)
SPDB P8 0-3 CM	Soil 1	A	54.31	1.34×10^{3}	0.0204	0.0090	44.1	0.0124	0.0071	57.4	0.0103	0.0080	0.0059	74.1776	0.0048	0.18	0.17
PCT-1 3-6 CM	Soil 2	A	50.02	1.23×10^{3}	0.0551	0.0050	9.1	0.0354	0.0036	10.3	0.0033	0.0197	0.0032	16.4353	0.0015	0.15	0.03
PCB-1 33-36 CM	Soil 3	A	41.27	1.11×10^{3}	0.1347	0.0130	9.7	0.0889	0.0121	13.6	0.0042	0.0458	0.0041	8.9182	0.0019	0.14	0.02
PDMT 6-9 CM	Soil 4	A	53.52	1.47×10^{3}	< DL			< DL			0.0351	0.0205	0.0108		0.0136	< DL	
PDM P8 9-12 CM	Soil 5	A	59.43	1.81×10^{3}	0.0446	0.0070	15.7	0.0300	0.0062	20.5	0.0076	0.0146	0.0031	21.1977	0.0035	0.13	0.04
SPDB P8 0-3 CM	Soil 1	B	96.02	1.57×10^{3}	0.0237	0.0060	25.3	0.0142	0.0036	25.3	0.0087	0.0095	0.0049	51.9047	0.0040	0.18	0.11
PCT-1 3-6 CM	Soil 2	B	65.91	2.91×10^{3}	0.0600	0.0060	10.0	0.0364	0.0051	14.2	0.0024	0.0237	0.0027	11.2773	0.0011	0.18	0.03
PCB-1 33-36 CM	Soil 3	B	69.97	1.96×10^{3}	0.1216	0.0050	4.1	0.0780	0.0046	5.9	0.0045	0.0436	0.0020	4.5148	0.0010	0.15	0.01
PDMT 6-9 CM	Soil 4	B	63.67	2.14×10^{3}	0.0570	0.0090	15.8	0.0378	0.0064	16.9	0.0088	0.0191	0.0063	33.0949	0.0035	0.14	0.05
PDM P8 9-12 CM	Soil 5	B	80.77	5.08×10^{3}	0.0403	0.0060	14.9	0.0266	0.0047	17.5	0.0046	0.0137	0.0037	26.9369	0.0014	0.14	0.04
SPDB P8 0-3 CM	Soil 1	C	55.97	1.76×10^{3}	< DL			< DL			0.0230	< DL			0.0212	< DL	
PCT-1 3-6 CM	Soil 2	C	60.56	9.47×10^{2}	< DL			< DL			0.0231	< DL			0.0223	< DL	
PCB-1 33-36 CM	Soil 3	C	48.31	9.40×10^{2}	0.1116	0.0229	20.5	0.0821	0.0176	21.4	0.0266	0.0295	0.0147	49.8232	0.0259	0.10	0.05
PDMT 6-9 CM	Soil 4	C	51.79	1.53×10^{3}	< DL			< DL			0.0260	< DL			0.0238	< DL	
PDM P8 9-12 CM	Soil 5	C	31.83	1.30×10^{3}	< DL			< DL			0.0429	< DL			0.0410	< DL	
SPDB P8 0-3 CM	Soil 1	D	44.59	4.92×10^{3}	0.0198	0.0057	29.0	0.0149	0.0041	27.7	0.0094	0.0049	0.0040	81.4683	0.0025	0.09	0.08
PCT-1 3-6 CM	Soil 2	D	51.89	3.64×10^{3}	0.0644	0.0067	10.3	0.0418	0.0060	14.3	0.0077	0.0226	0.0030	13.1737	0.0019	0.15	0.03
PCB-1 33-36 CM	Soil 3	D	55.83	9.43×10^{3}	0.1034	0.0075	7.2	0.0672	0.0048	7.2	0.0069	0.0362	0.0057	15.6701	0.0017	0.15	0.02
PDMT 6-9 CM	Soil 4	D	50.02	3.86×10^{3}	0.0631	0.0068	10.7	0.0404	0.0064	15.9	0.0052	0.0227	0.0022	9.6000	0.0033	0.15	0.03
PDM P8 9-12 CM	Soil 5	D	51.86	4.18×10^{3}	0.0449	0.0049	11.0	0.0319	0.0035	10.9	0.0041	0.0130	0.0035	26.7316	0.0029	0.11	0.03
SPDB P8 0-3 CM	Soil 1	E	71.07	1.27×10^{4}	0.0334	0.0036	10.7	0.0240	0.0026	10.9	0.0024	0.0094	0.0024	25.7844	0.0008	0.11	0.03
PCT-1 3-6 CM	Soil 2	E	71.85	6.29×10^{3}	0.0718	0.0058	8.1	0.0471	0.0055	11.7	0.0018	0.0246	0.0018	7.4617	0.0004	0.14	0.02

PCB-1 33-36 CM	Soil 3	E	63.84	8.46×10^{3}	0.1398	0.0119	8.5	0.0922	0.0111	12.1	0.0021	0.0477	0.0043	9.0173	0.0004	0.14	0.02
PDMT 6-9 CM	Soil 4	E	59.82	1.92×10^{4}	0.0647	0.0040	6.2	0.0429	0.0024	5.6	0.0023	0.0218	0.0032	14.8522	0.0006	0.14	0.02
PDM P8 9-12 CM	Soil 5	E	79.37	1.78×10^{4}	0.0457	0.0029	6.2	0.0298	0.0023	7.7	0.0050	0.0159	0.0017	10.5525	0.0019	0.15	0.02
SPDB P8 0-3 CM	Soil 1	FD	84.12	1.07×10^{4}	0.0249	0.0050	20.2	0.0163	0.0039	23.8	0.0085	0.0087	0.0032	36.9119	0.0059	0.15	0.06
PCT-1 3-6 CM	Soil 2	FD	84.16	9.00×10^{3}	0.0599	0.0059	9.8	0.0389	0.0032	8.2	0.0082	0.0210	0.0049	23.5495	0.0059	0.15	0.04
PCB-1 33-36 CM	Soil 3	FD	87.98	9.09×10^{3}	0.1183	0.0139	11.8	0.0769	0.0136	17.7	0.0078	0.0414	0.0031	7.5185	0.0057	0.15	0.03
PDMT 6-9 CM	Soil 4	FD	85.42	1.16×10^{4}	0.0656	0.0061	9.3	0.0433	0.0037	8.5	0.0080	0.0223	0.0049	22.0108	0.0057	0.14	0.03
PDM P8 9-12 CM	Soil 5	FD	80.71	9.80×10^{3}	0.0514	0.0091	17.7	0.0306	0.0069	22.4	0.0089	0.0208	0.0060	28.6806	0.0063	0.19	0.07

Table S2. Results of ashing experiment.

Sample	Ashing Temperature (°C)	239+240Pu (Bq/kg)	Uncertainty (Bq/kg)
Soil A	450	0.067	0.003
Soil A	600	0.063	0.003
Soil A	1000	0.013	0.002
Soil B	450	0.006	0.002
Soil B	600	< DL	-
Soil B	1000	< DL	-
Soil C	450	0.125	0.008
Soil C	600	0.127	0.004
Soil C	1000	0.021	0.002
Soil D	450	0.075	0.005
Soil D	600	0.081	0.006
Soil D	1000	0.026	0.003
Soil E	450	< DL	-
Soil E	600	0.019	0.004
Soil E	1000	< DL	-
Soil F	450	0.144	0.006
Soil F	600	0.126	0.004
Soil F	1000	0.048	0.004

Table S3. Means of spike recovery percentages and ^{238}U separation factors for the different procedures (Methods A-E) and fusion digestion procedure (Method FD).

Method	^{242}Pu recovery (%)	SD	^{238}U separation factor	SD
A	51.71	6.73	1.39×10^3	0.27×10^3
B	75.27	13.33	2.73×10^3	1.40×10^3
C	49.69	10.99	1.30×10^3	0.36×10^3
D	50.83	4.08	5.21×10^3	2.41×10^3
E*	69.19	7.59	1.29×10^4	0.56×10^4
FD	84.48	2.63	1.00×10^4	0.11×10^4

*PULSE method

Table S4. IAEA-412 certified and method-obtained values for Pu isotopes.

	Plutonium isotopes							
	^{239}Pu (Bq/kg)	Unc (Bq/kg)	^{240}Pu (Bq/kg)	Unc (Bq/kg)	$^{239+240}Pu$ (Bq/kg)	Unc (Bq/kg)	$^{240}Pu/^{239}Pu$ (at ratio)	Unc (at ratio)
IAEA-412 Certified Values	0.358	0.016	0.240	0.016	0.611	0.028	0.182	0.015
PULSE method	0.386	0.016	0.24	0.012	0.644	0.020	0.183	0.007

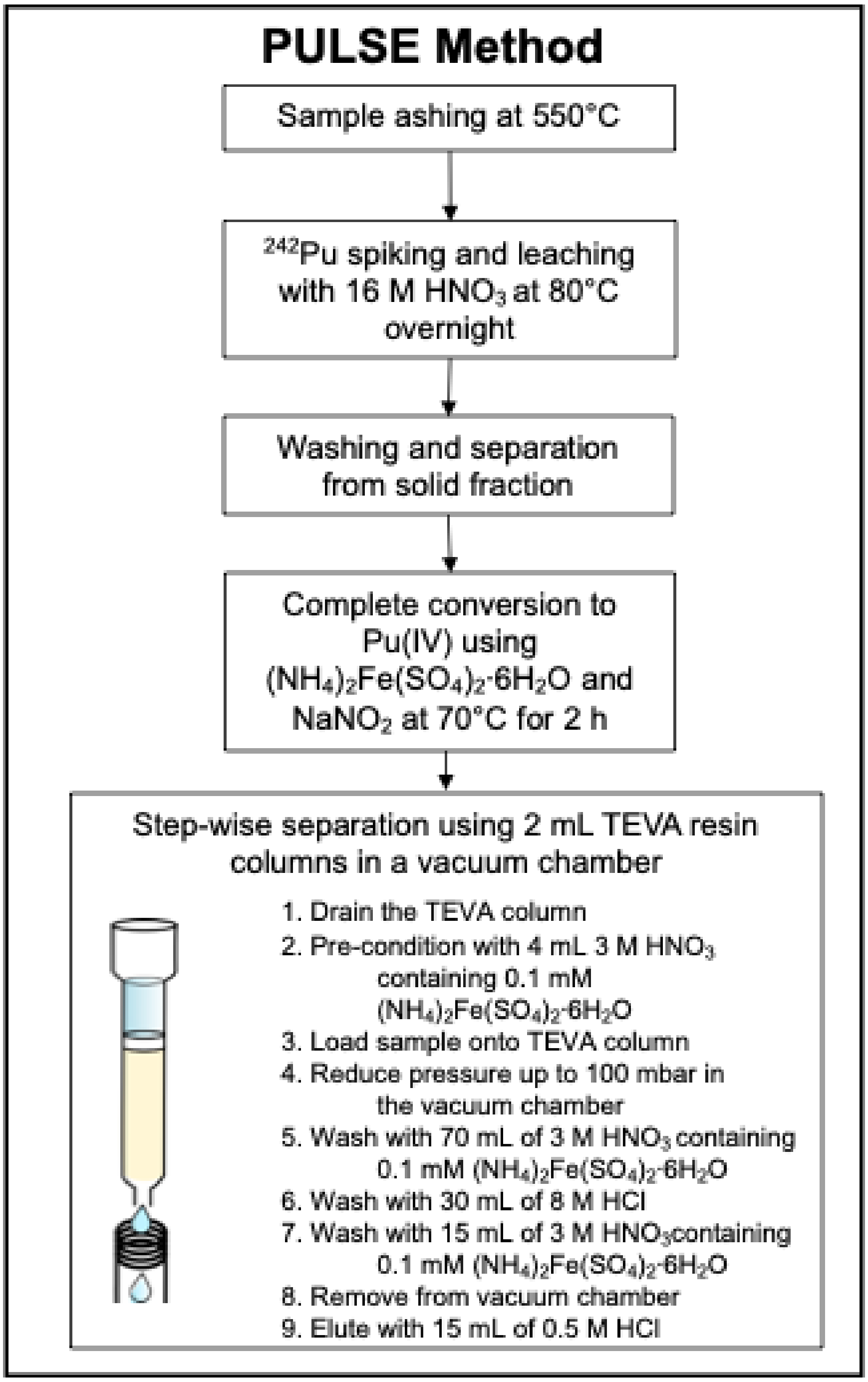

Figure S1. Flowchart of the optimal modified leaching and separation procedure.
